# Hydrogen sulfide and its role in female reproduction

**DOI:** 10.3389/fvets.2024.1378435

**Published:** 2024-06-12

**Authors:** Aneta Pilsova, Zuzana Pilsova, Barbora Klusackova, Natalie Zelenkova, Eva Chmelikova, Pavla Postlerova, Marketa Sedmikova

**Affiliations:** Department of Veterinary Sciences, Faculty of Agrobiology, Food, and Natural Resources, Czech University of Life Sciences Prague, Prague, Czechia

**Keywords:** hydrogen sulfide, female reproduction, cystathionine beta synthase, cystathionine gamma lyase, oocyte physiology, early embryo development, uterus, gravidity

## Abstract

Hydrogen sulfide (H_2_S) is a gaseous signaling molecule produced in the body by three enzymes: cystathionine-β-synthase (CBS), cystathionine-γ-lyase (CSE) and 3-mercaptopyruvate sulfurtransferase (3-MST). H_2_S is crucial in various physiological processes associated with female mammalian reproduction. These include estrus cycle, oocyte maturation, oocyte aging, ovulation, embryo transport and early embryo development, the development of the placenta and fetal membranes, pregnancy, and the initiation of labor. Despite the confirmed presence of H_2_S-producing enzymes in all female reproductive tissues, as described in this review, the exact mechanisms of H_2_S action in these tissues remain in most cases unclear. Therefore, this review aims to summarize the knowledge about the presence and effects of H_2_S in these tissues and outline possible signaling pathways that mediate these effects. Understanding these pathways may lead to the development of new therapeutic strategies in the field of women’s health and perinatal medicine.

## GRAPHICAL ABSTRACT

BioRender was used to create images and diagrams.

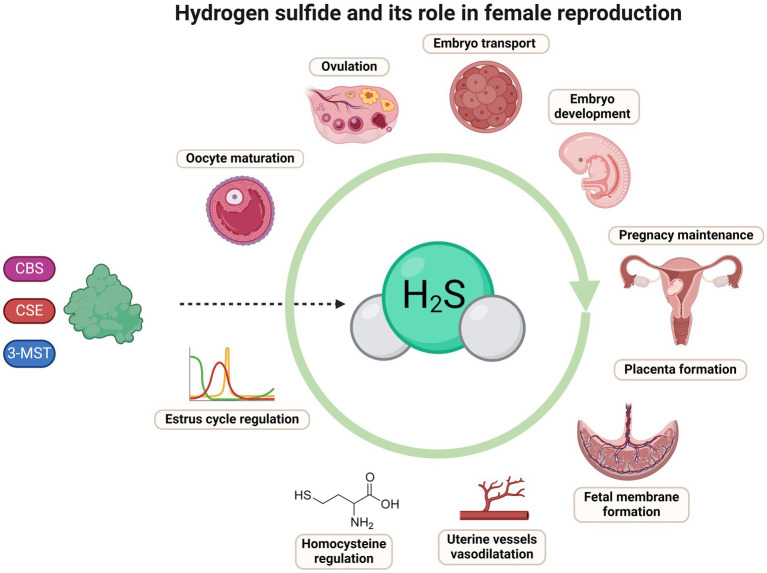

## 1 Introduction

Several decades ago, hydrogen sulfide was considered only as a toxic gas. However, after the discovery of endogenous production of nitric oxide (NO) (1) and carbon monoxide (CO) (2) in the organism and their effects on various tissues, a third endogenously produced gasotransmitter, hydrogen sulfide (H_2_S), was demonstrated (3). H_2_S is now known to be involved in a wide range of physiological processes, including reducing cellular oxidative stress, regulating the cell cycle and apoptosis, participating in inflammatory processes, and vasodilating blood vessels (4). The regulation of the nervous and reproductive systems are among the other described functions of H_2_S (4, 5).

**Figure 1:**
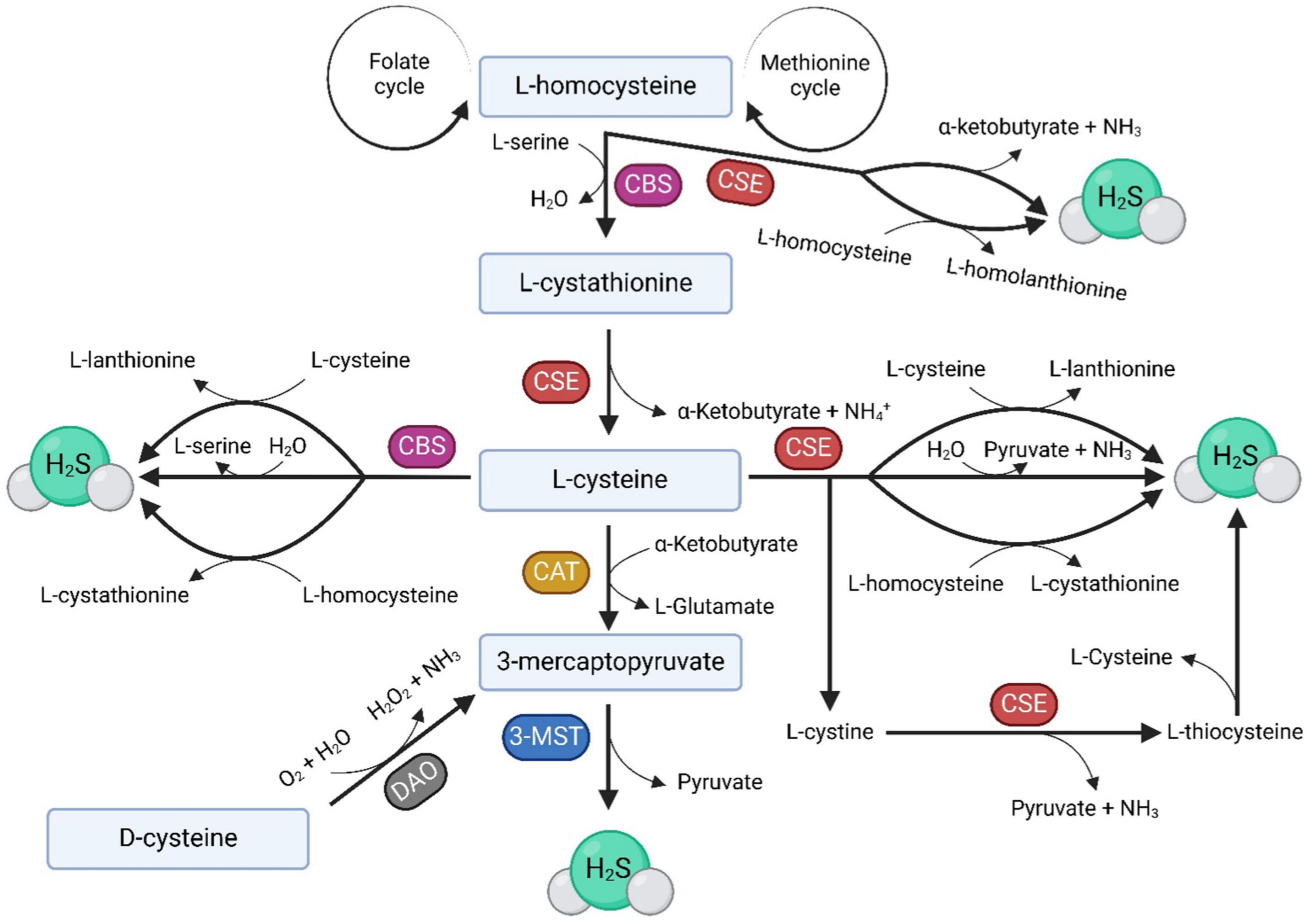
Anabolic pathways of H_2_S. H_2_S is enzymatically produced in the body by three enzymes: CBS, CSE, and 3-MST, which also requires CAT for H_2_S production. The main substrate for H_2_S formation is L-cysteine, which can, under the influence of H_2_S-producing enzymes, be produced from homocysteine (Hcy) supplied by the folate and methionine cycles. The image illustrates various pathways involved in the endogenous production of H_2_S in the body under the influence of CBS, CSE, and 3-MST, as well as the byproducts of these biochemical reactions.

Three enzymes are responsible for the endogenous production of H_2_S, namely cystathionine-β-synthase (CBS), cystathionine-γ-lyase (CSE) and 3-mercaptopyruvate sulfurtransferase (3-MST). In addition to these, cysteine aminotransferase (CAT) is sometimes mentioned as the fourth H_2_S-producing enzyme (Figure 1) (6, 7). The main substrate for the enzymatic production of H_2_S is L-cysteine (8) (Figure 1), although physiologically, H_2_S can also be generated from D-cysteine (9). However, H_2_S can also be produced through non-enzymatic processes, such as its production by microorganisms in the digestive tract that metabolize sulfur or the simple dissociation of sodium hydrosulfide (NaHS) into H_2_S. H_2_S can also be released from acid-labile sulfur, which serves as a reservoir of this molecule in the body (10, 11).

One of the many organ systems affected by H_2_S is the reproductive tract. H_2_S has been detected in both male and female reproductive tracts of mammals, fish, and amphibians. In the male reproductive tract, one of the most fundamental roles of H_2_S is the facilitation of erection (12, 13). In the female reproductive system, H_2_S has been detected in oocytes (14), follicular cells at all stages (15), the uterus (16), and the placenta (17). In female reproduction, H_2_S is essential during gravidity and labor initiation (17, 18), in oocyte maturation and ovulation (19). It also influences the vasodilation of uterine and placental vessels, thereby affecting the nutrition of the growing embryo/fetus, in whose epigenetic regulation H_2_S also participates (20, 21). H_2_S production also occurs in the vagina and clitoral smooth muscle, where it supports smooth muscle relaxation, vaginal lubrication, and epithelial ion transport (22).

## 2 Molecular targets of H_2_S

**Figure 2:**
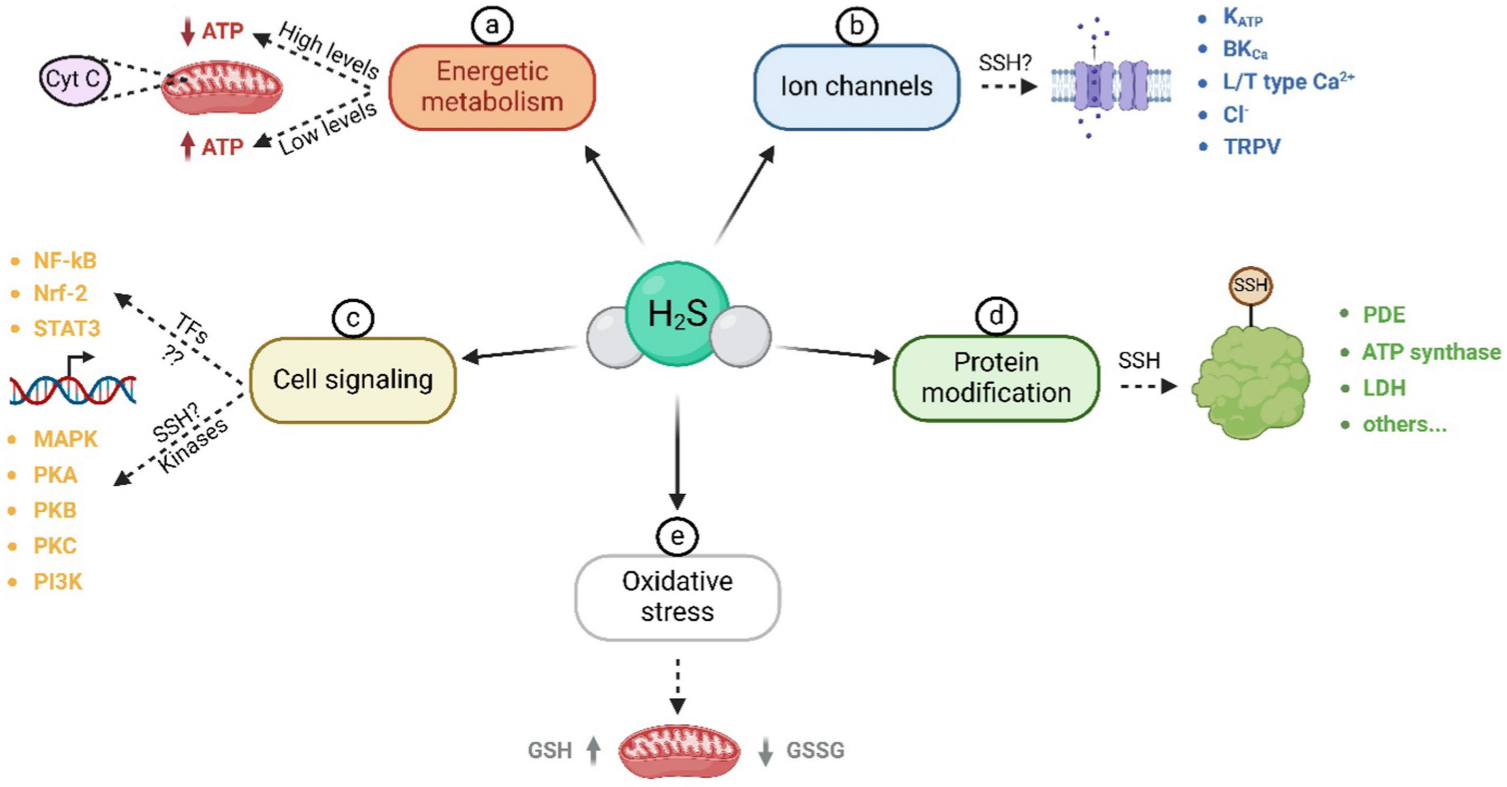
Molecular targets of H_2_S. These targets can be divided into the following groups: a) Influence of H_2_S on energy metabolism and cytochrome c oxidase activity; b) activation and inactivation of various types of ion channels, likely through S-sulfhydration; c) influence on cell signaling through transcription factors and kinases; d) modification of a wide range of proteins through S-sulfhydration of cysteine thiol sites; e) reduction of oxidative stress in mitochondria. ATP – adenosine triphosphate; SSH – S-sulfhydration; NF-κB – nuclear factor-kappa-B; Nrf-2 – nuclear factor E2-related factor 2; STAT3 – signal transducer and activator of transcription 3; MAPK – mitogen-activated protein kinase; PKA – protein kinase A; PKB – protein kinase B; PKC – protein kinase C; PI3K – phosphoinositide 3-kinase; PDE – phosphodiesterase; LDH – lactate dehydrogenase; GSH – glutathione; GSSG – glutathione disulfide.

The effects of H_2_S on various molecular targets are summarized in Figure 2.The first confirmed target of H_2_S was cytochrome c oxidase in mitochondria. In high H_2_S concentrations, mitochondrial activity can be inhibited, and thus adenosine triphosphate (ATP) production is prevented. However, in lower concentrations, H_2_S can supply electrons to the mitochondrial respiratory chain through sulfide quinone oxidoreductase and cytochrome c oxidase (23, 24). In mitochondria, there has been detected the H_2_S-producing enzyme – 3-MST (25, 26). H_2_S is associated with cellular oxidative stress, as it interacts with glutathione, leading to an elevation in its concentration and the subsequent suppression of oxidative stress in mitochondria (27, 28).

Transcription factors are other H_2_S intracellular targets during inflammatory processes, as well as during embryonic development. H_2_S donors such as NaHS, S-diclofenac, or diallyl sulfide can inhibit nuclear factor kappa B (NF-κB) activation, thereby suppressing the production of pro-inflammatory cytokines (29). Conversely, under certain conditions (dose, exposure time), H_2_S may have pro-inflammatory effects in NF-κB in/dependent manner (30). Both results point to the influence of H_2_S on inflammatory processes and its tissue specificity. H_2_S likely impacts other transcription-mediated processes, such as proliferation (31) or angiogenesis (32), and it appears to play a crucial role in the epigenetic regulation of genes in early embryos (33).

A variety of kinases are also cellular targets of H_2_S. Examples are mitogen-activated protein kinases (MAPK), which H_2_S can both activate (19, 34) through S-sulfhydration (35) and inhibit (36, 37). H_2_S also activates protein kinase A (PKA) (38, 39), phosphoinositide 3-kinase (PI3K)/protein kinase B (PKB) (40–42) or protein kinase C (PKC) (41). Additionally, to targeting kinases, H_2_S also inhibits phosphodiesterase, and consequently regulates the levels of cyclic guanosine monophosphate (cGMP) (43, 44).

Molecular targets of H_2_S that should be noticed are cellular proteins themselves. An important effect of H_2_S is the S-sulfhydration of proteins. This process involves the delivery of a sulfur atom derived from the H_2_S molecule to the thiol group of cysteine residues, leading to the formation of a hydropersulfide group (-SSH) (45, 46). These -SSH cysteines are more reactive than cysteines containing only a thiol group, and S-sulfhydration modifies these proteins (46). Interestingly, in cells, S-sulfhydration is considered a common post-translational modification. Among S-sulfhydrated proteins belong ATP synthase, lactate dehydrogenase, ion channels, phosphodiesterase, and many others (23, 44, 45, 47). In ion channels, H_2_S is capable of opening ATP-sensitive potassium channels (K_ATP_) in the smooth muscle of arteries (48), myocytes (49), and smooth muscle of the intestine (50) or eye (51). However, H_2_S also regulates other channels such as large-conductance calcium-activated potassium ion channels (BK_Ca_) (52), L-type and T-type Ca^2+^ channels (53, 54), Cl^−^ channels (55), and transient receptor potential vanilloid and ankyrin channels (TRPV and TRPA) (56, 57). S-sulfhydration can activate some channels while inhibiting others. Among activated channels belong K_ATP_ (58, 59), Cl^−^ (55), TRPV/TRPA (56, 57), T-type Ca^2+^ (60) and BK_Ca_ channels (52, 61). Inhibited ion channels via S-sulfhydration are L-type Ca^2+^ (45, 53, 62), T-type Ca^2+^ (54) and BK_Ca_ channels (63).

## 3 Detection of H_2_S-producing enzymes and the role of H_2_S in female reproductive tissues

Over the last two decades, H_2_S-producing enzymes have been detected in various female reproductive tract tissues, spanning different animal models, including humans. Table 1 provide summary of the experiments conducted on this topic across diverse animal species and describe the potential significance of H_2_S-producing enzymes in these tissues.

**Table 1:** Detection of H_2_S-producing enzymes in female reproductive tissues.

Tissue	Model	Enzyme	Findings	Source
Oocytes and follicular cells	Human	CBS	Expression in GV*, regulation of the assembly of the mitotic spindle	(14)
		CSE	Expansion of cumulus-oophorus (CO), regulation of ovulation	(82)
	Mouse	CBS	Expression in superovulated COC**, granulosa cells, nucleus, GV*, regulation of the assembly of the mitotic spindle	(14, 80)
		CSE	Expansion of CO, regulation of ovulation	(82)
	Pig	CBS CSE 3-MST	Influence of CO expansion, support of oocyte maturation	(19, 81)
	*Xenopus laevis*	CBS CSE 3-MST	Modulation of oocyte meiosis	(72)
Oviduct	Human	CBS	Relaxation of smooth muscle cells, promotion of embryo transport to the uterus	(83)
Uterus	Human	CBS CSE	Decreased expression with the onset of labor	(16, 18, 114)
	Mouse	CBS CSE	Increased expression during estrus and diestrus	(64, 114, 130, 140, 142)
	Rat	CBS	Decreased activity during gravidity	(132)
Uterine vessels	Human	CBS	Uterine artery vasodilatation regulated by E2	(98)
		CSE 3-MST	Uterine artery vasodilatation	(20)
	Sheep	CBS CSE	Uterine artery vasodilatation regulated by E2	(98)
Placenta	Human	CBS CSE	Decreased expression during labor	(17, 133, 140, 163)
	Rat	CBS	Decreased activity during gravidity	(132)
Fetal membranes	Human	CBS	Regulation of early embryonic development	(18, 33)
		CSE	Regulation of early embryonic development, regulation of vasomotor tone in the fetoplacental vasculature	(18, 33, 163)
	Rat	CBS CSE	Regulation of early embryonic development	(18)
Embryo	Human	CBS CSE	Promotion of placental angiogenesis, regulation of early embryonic development	(33, 140, 143)
	Zebrafish	CBS	Influence on the development of the anteroposterior axis	(158)
Umbilical vessels	Human	CBS CSE	Vasodilatation	(164)
Vaginal epithelium	Rabbit	CBS CSE	Relaxation of vaginal and clitoral smooth muscle	(120)
	Rat	CSE	Regulation of production and composition of vaginal fluid	(22)

*GV, germinal vesicle; **COC, umulus-oocyte complex.

**Figure 3:**
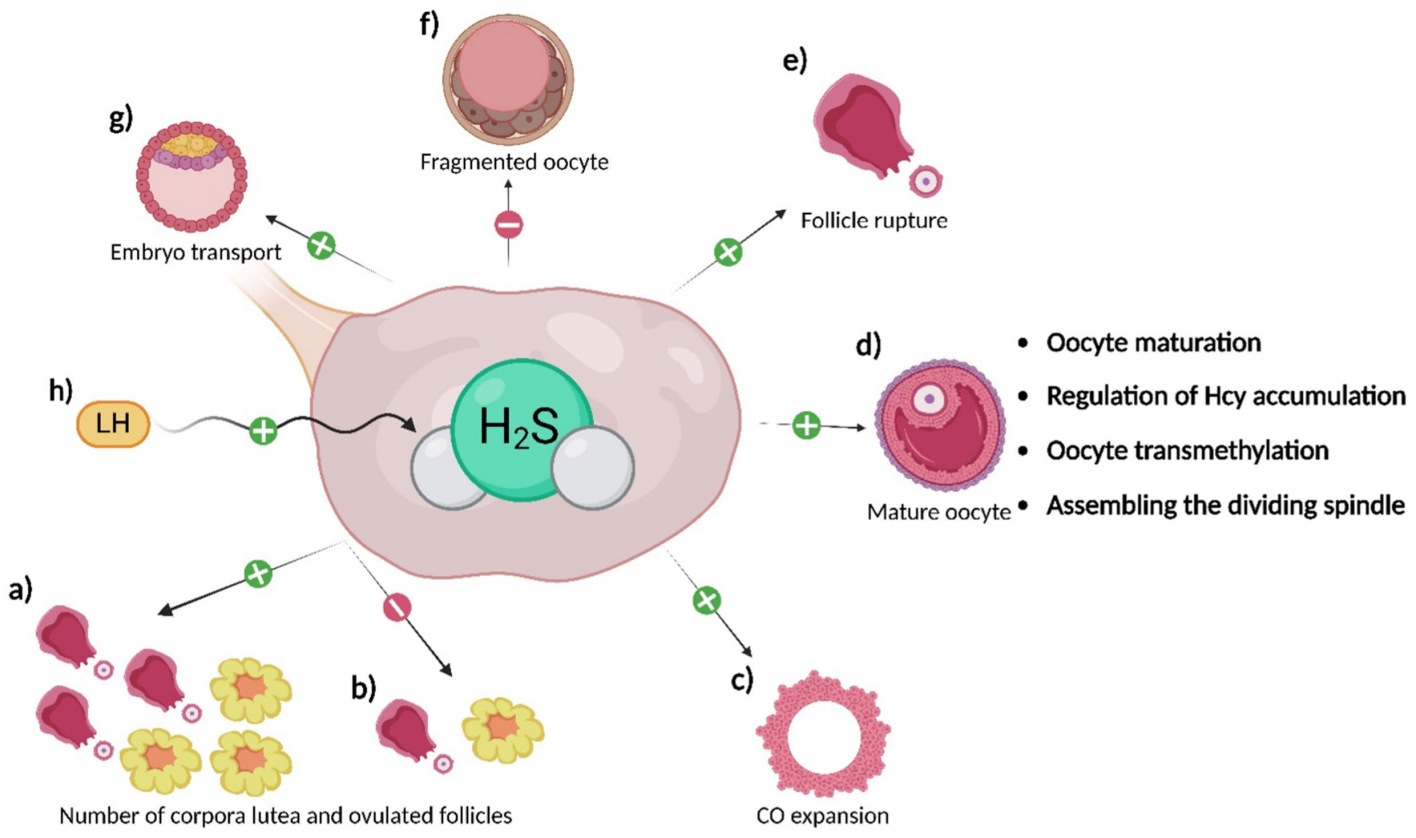
Effects of H_2_S on oocytes. H_2_S produced in the ovaries and surrounding reproductive tissues has the following effects: a) H_2_S promotes ovulation of a more significant number of follicles and the development of a greater number of corpora lutea compared to inhibiting its production (b); c) H_2_S supports CO expansion; d) H_2_S promotes oocyte maturation through the regulation of oocyte signaling pathways. Additionally, its production regulates Hcy levels, supports oocyte transmethylation, and ensures proper spindle assembly; e) H_2_S promotes follicle rupture and, therefore, oocyte ovulation itself; f) H_2_S delays oocyte aging and reduces the number of fragmented oocytes; its inhibition, on the other hand, leads to an increase in the number of fragmented oocytes; g) H_2_S supports embryo transport to the uterus; h) LH stimulates H_2_S production in granulosa cells of follicles, likely through CSE, contributing to the processes mentioned above.

Among the initial experiments investigating the function of H_2_S in the female reproductive system, knockout studies (*CBS-KO*; *CSE-KO*) have been described (64–67). These studies demonstrate the importance of CBS in the maintenance of placental and uterine weight in females, as well as its indispensability in the maturation of growing follicles (64, 65) (Figure 3). Furthermore, the effect of CBS on the regularity and length of the estrus cycle was proven, which subsequently affects the fertility rate in females (64, 68). However, the absence of CBS does not cause morphological abnormalities on ovulated oocytes or the ovaries themselves (64). Interestingly, after transplanting *CBS-KO* ovaries into healthy recipients, the fertility of the females was not affected, indicating that the H_2_S production through CBS in other reproductive tissues is sufficient but probably not essential for maintaining female fertility (64). As for CSE, the absence of this H_2_S-producing enzyme in mice appears to have significantly less effect on the incidence of fertility-related defects, as *CSE-KO* females were fertile, and their pregnancies progressed normally (65, 67, 69). Recent research focused on the fertility of *CSE-KO* mice showed that *CSE-KO* leads to a reduced number of successful pregnancies and a higher pro-inflammatory status of fetuses. This suggests that CBS is not the sole key enzyme in H_2_S production in the context of female reproduction (70).

The reason why CBS seems more important for female reproduction in most studies (64, 65, 67) when the final product of both enzymes is H_2_S, has yet to be investigated. Potential reasons may include variances in homocysteine (Hcy) and cysteine metabolic pathways or differences in the substrate essential for the H_2_S formation. CBS utilizes Hcy or L-cysteine for H_2_S production (71, 72), with L-cysteine also generated from Hcy by both CBS and CSE (Figure 1). In reproductive tissues, the prevalence of Hcy may favor CBS (18, 73). CSE primarily uses cystathionine/L-cysteine/cystine as a substrate, but it can also utilize Hcy (74–77). However, the direct production of H2S from Hcy by CSE suggests a potential advantage in following the CBS route, interrupting the reaction at the intermediate product, cystathionine, to regulate both Hcy and H2S levels in the body (Figure 1). This proposition is supported by the fact that hyperhomocysteinemia is a critical factor during pregnancy leading, for example, to preeclampsia, miscarriages, uterine artery blood flow resistance or congenital malformations (73, 76, 78). Furthermore, higher H_2_S levels can lead to the inhibition of cytochrome c oxidase in mitochondria (24, 79). It is possible that CBS was evolutionarily favored because it can effectively regulate both Hcy levels in tissues and the H_2_S levels. However, further experiments are necessary to understand CBS’s role in female reproduction precisely.

### 3.1 The role of H_2_S in oocytes

The influence of H_2_S on oocyte maturation (19, 80, 81), ovulation (15, 82), and embryo transport to the uterus (83) has been studied in mice and human oocytes, particularly in connection with luteinizing hormone (LH), which increases CSE production in granulosa cells (82). Inhibition of CSE leads to a reduced number of ovulating follicles and corpus luteum and a higher number of unovulated follicles with retained oocytes (64, 65, 82). LH likely stimulates H_2_S production in granulosa cells in the preovulatory period (82). Furthermore, the regulation of H_2_S through a donor increased the levels of proteins essential for cumulus-oophorus (CO) expansion and follicle rupture (82, 84, 85). These results highlight the connection between the hormonal regulation of female reproduction and H_2_S production (68).

Regarding the role of H_2_S in oocyte maturation, it has been hypothesized that CBS acts as a mediator between the oocyte and granulosa cells and it may contribute to the proper flow of Hcy in follicular cells, and subsequently support the stability of oocyte transmethylation (15, 80). H_2_S plays a role during oocyte maturation in the intracellular environment of the oocyte as well. H_2_S regulates signalling pathways during the cell cycle, likely through S-sulfhydration (35). As was mentioned above, H_2_S-mediated regulation has been described in the cAMP-PKA, PI3K-PKB, MAPK and maturation promoting factor (MPF) pathways (43, 46, 72, 84). Using H_2_S donor (Na_2_S), the supporting effect of the H_2_S on oocyte maturation has been proven, as the Na_2_S accelerated the porcine oocyte nuclear maturation and increased MPF activity during GVBD stage. Moreover, this donor increased the number of zygotes with formed pronuclei after the parthenogenetic activation of porcine oocytes (81, 84, 85). During the germinal vesicle stage (GV), CBS is distributed into the nucleus of oocytes, however, from germinal vesicle breakdown (GVBD) to metaphase II, it is localized around the mitotic spindle, where it is probably essential for acetylation of α-tubulin and proper assembly of the mitotic spindle (Figure 3). Conversely, deletion of the CBS gene leads to meiosis arrest, abnormalities in both the meiotic spindle and chromosome structure and disruption of the kinetochore-microtubule attachment (14). Additionally, H_2_S is produced by cumulus cells, which likely promotes CO expansion (19, 86). The importance of H_2_S during oocyte maturation is further supported by the findings of Gelaude et al. (72), who confirmed the effect of H_2_S on meiosis in amphibian oocytes.

It has been previously described that H_2_S has anti-aging effects and promotes the longevity, health, and condition of many organ systems, including the fetal membranes, probably through the mammalian target of rapamycin (mTOR) signaling pathway and its downstream factor S6 kinase beta-1 (S6K1) (87–89). For this reason, a series of experiments describing the role of H_2_S during oocyte aging have been reported. H_2_S-producing enzymes are active in porcine oocytes, and there is a statistically significant decrease in endogenous H_2_S production during the first day of aging. Inhibition of H_2_S-producing enzymes induces signs of aging in oocytes and significantly increases the number of fragmented oocytes (Figure 3)(90). Conversely, an exogenous H_2_S donor (Na_2_S) can reverse these manifestations. Cultivation in the presence of the H_2_S donor can also positively affect subsequent embryonic development after parthenogenetic activation (90). These results were supported by research confirming reduced CBS expression in oocytes and ovaries of old mice (14). The mechanism of H_2_S action on oocytes involves the regulation of K_ATP_ and L-type Ca^2+^ channels, which play a crucial role during oocyte aging through S-sulfhydration. H_2_S activates K_ATP_ channels, delaying cell death, and conversely inhibits L-type Ca^2+^ channels, which have the opposite effect on oocytes (45, 53, 62, 91). In conclusion, H_2_S is crucial in most processes occurring in oocytes (Figure 3) and their immediate environment.

### 3.2 The role of H_2_S in uterine tissues

#### 3.2.1 Uterine vessels

**Figure 4:**
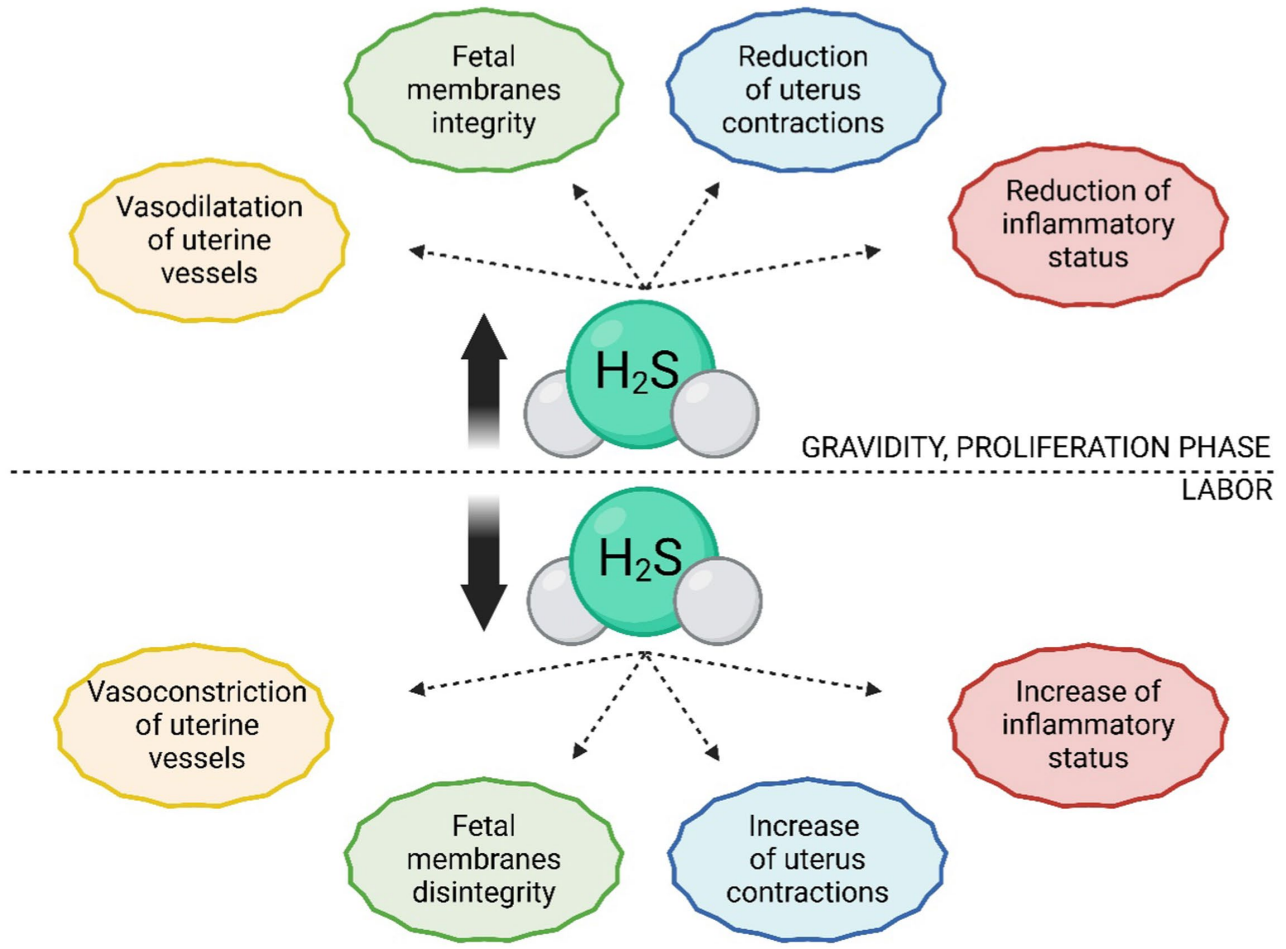
Comparison of H_2_S levels and their functions during pregnancy and labor. In estrogen-dominant phases, such as pregnancy or the proliferative phase of the estrus/menstrual cycle, there is an increased production of H_2_S. In uterine tissues, H_2_S promotes dilation of uterine blood vessels, contributing to tissue perfusion and proper development of the placenta, fetus, and nutrient supply to the fetus. It also maintains the integrity of fetal membranes and has tocolytic effects on the uterus, thereby delaying labor. Before and during labor, there is a significant reduction in H_2_S in uterine tissues, leading to constriction of uterine blood vessels, rupture of fetal membranes, and increased uterine contractions.

Given the vasodilatory effects of H_2_S (58, 92, 93), this function has been investigated concerning the regulation of blood flow in uterine vessels, which affects the exchange of nutrients and respiratory gases between the mother and the fetus, consequently influencing fetal growth and health (94, 95). It appears that the activity of H_2_S-producing enzymes and the subsequent effect of uterine blood vessel vasodilation are essential, as elevated levels of Hcy (and thus a probable deficiency in H_2_S-producing enzymes) lead to uterine artery blood flow resistance (96). Vasodilatory effects of H_2_S have been confirmed in human (97), sheep (98), and rat (99) uterine arteries, as well as in human umbilical arteries and veins, with this effect occurring primarily during the proliferative phase of the menstrual (estrus) cycle and in gravidity (Figure 4) (20). The mechanism of vasodilation in the vascular system generally occurs through K_ATP_ channels (62, 100, 101). The same mechanism is employed in uterine vessels, as was confirmed by subsequent studies describing an increased number of K_ATP_ channels in human and sheep smooth muscle cells of uterine arteries during pregnancy (20, 102). However, Li et al. (103) contributed to this topic by elucidating the regulation of BK_Ca_ channels by H_2_S in human uterine arteries, so it is conceivable that multiple types of ion channels contribute to the vasodilation of uterine arteries by H_2_S.

In the past decade, studies have emerged reporting the regulation of uterine vessel vasodilation by estrogens through their influence on promoting H_2_S synthesis via CBS and CSE (104). For example, it has been described that during estrogen-dominant phases of the female cycle (i.e., proliferation, pregnancy), CBS production is higher than the secretory phase. Specifically, CBS seems to be the primary H_2_S-producing enzyme responding to elevated estrogen levels, as the expression of CSE and 3-MST does not change in gravid tissue compared to non-gravid tissue (20, 105). Interestingly, Zeigler et al. (106) found a decrease in plasma H_2_S levels in the later stages of pregnancy compared to postpartum. This could be explained more likely as an increase in H_2_S consumption, as it is essential for S-sulfhydration of proteins necessary for the growth of maternal and fetal tissues. S-sulfhydrated proteins are extensively involved in processes such as the contraction and relaxation of smooth muscle in blood vessels (107, 108). Additionally, during pregnancy, the H_2_S dilution is more significant as the volume of maternal blood plasma can increase by up to 50% (106, 109). This hypothesis is supported by the increased production of H_2_S in intrauterine tissues during pregnancy, which leads to a higher rate of S-sulfhydration of proteins compared to non-pregnant tissue. These results support the finding that the expression of CBS is greater in estrogen-dominant phases, as the consumption of H_2_S is also higher (110).

While the precise mechanism describing estrogen-induced stimulation of H_2_S biosynthesis in uterine arteries is unknown, a hypothesis suggests estrogen receptors’ important role in this signaling pathway (104). This hypothesis has been recently confirmed, as it was found that estrogen receptors activate CBS promoters, thereby stimulating its production. In contrast, the activity of the CSE promoter remains unchanged (110). When it comes to vascular dilation, it is worth noting the previously established influence of another gasotransmitter – NO, which is also a potent vasodilator in the bloodstream and interacts with H_2_S in many organ systems (111–113). It is presumable that H_2_S, NO, and estrogens, which interact with both H_2_S and NO, synergistically contribute to the vasodilation of uterine vessels and that these systems behave towards each other as backup mechanisms because pathology occurs only after the inhibition of both signaling pathways (111, 113).

#### 3.2.2 Uterus and pregnancy

**Figure 5:**
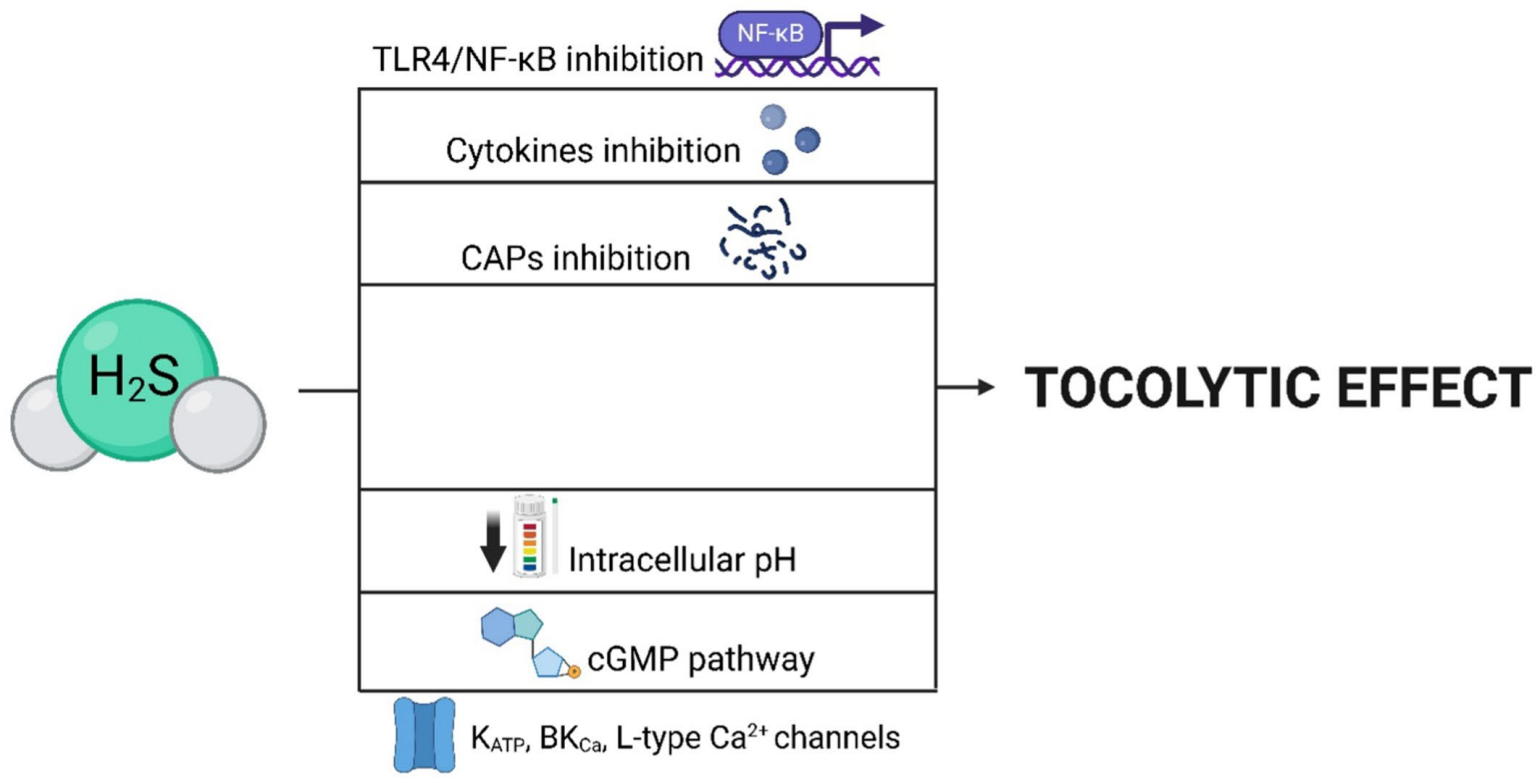
Mechanisms leading to the tocolytic effect of H_2_S. The tocolytic effects of H_2_S can be mediated by the opening of ion channels (K_ATP_, BK_Ca_, L-type Ca^2+^), as well as through the cGMP pathway or a reduction in intracellular pH. H_2_S achieves these effects by inhibiting CAPs, pro-inflammatory cytokines, and the TLR4/NF-κB signaling pathway.

One of the most referred impacts of H_2_S on the human (114) and rat (115) uterus is its tocolytic effects, which can be caused by H_2_S itself, as well as its precursor (L-cysteine) (116) or donor (NaHS) (117). These effects, promoting uterine relaxation, are significant for gravidity maintenance. Therefore, it is not surprising that the expression of CBS and CSE and production of H_2_S increases during gravidity and, conversely, abruptly decreases with the onset of labor (110, 114, 118). H_2_S also effectively prolongs the duration of labor and reduces the frequency of uterine contractions, which can contribute to a smooth delivery process (Figure 4) (119). It is assumed that the mechanism of the tocolytic effects of H_2_S lies in the opening of channels, as the body utilizes the exact mechanism in the bloodstream and other smooth muscle tissues (18, 120). Additionally, it has been demonstrated that the inhibition of K_ATP_ channels leads to the absence of relaxation effects of H_2_S donors (45, 92). It is possible that H_2_S also regulates activity of BK_Ca_ channels and L-type Ca^2+^ channels, as they also influence the relaxation of myometrium (52, 73, 118). Furthermore, the tocolytic effects of H_2_S may lie in inhibition of contraction-associated proteins (CAPs) and suppressing the toll-like receptor 4 (TLR4)/NF-κB signaling pathway (Figure 5) (16, 29, 42). Besides its tocolytic effects, H_2_S may also impact uterine immune response and placental vessel remodeling through the modulation of the uterine natural killer (uNK) cells (121–123). H_2_S signaling is also essential for maintaining early pregnancy, and its deficiency can lead to reduced litter size due to early embryo loss or placental inflammation (70, 124). H2S may further facilitate the physiological implantation of the embryo by regulating ion transport activity in the endometrial epithelium and supporting DNA synthesis (125–127).

The relationship between H_2_S and estradiol (E2) is intriguing because both contribute to uterine quiescence during pregnancy by regulating the expression of CAPs (128, 129). Estrogens, in general, appear to regulate H_2_S-producing enzymes, consequently affecting the levels of H_2_S itself (98). The increased production of CBS and H_2_S in the uterine arteries during pregnancy is influenced by endogenous estrogens acting through specific estrogen receptors (ER) in pregnant rats. This indicates that the physiological changes associated with pregnancy, such as elevated levels of endogenous estrogens, play a role in stimulating the expression of CBS and subsequent H_2_S production in the uterine arteries. The specific ER-mediated mechanism implies that ER are involved in regulating this process, highlighting the importance of endogenous estrogen signaling in mediating vascular adaptations during pregnancy (110). Specifically, E2 modulates gene expression and redox balance in the uterus by inducing transsulfuration via CBA and CSE, for which this metabolic pathway is unique (20, 130). The effects of E2 may also influence the metabolism of myometrial cysteine, which is utilized by H_2_S-producing enzymes to generate H_2_S, particularly during periods of elevated E2 levels such as estrus and gravidity. This pathway is mediated through sulfur amino acids and myometrial cysteine sulfinic acid decarboxylase (CSAD), the activity of which is reduced by E2. Estrogen-mediated regulation of H_2_S-producing enzymes and H_2_S itself occurs not only in uterine tissue but also in uterine vessels, where E2 activates CBS promotors leading to increased production of H_2_S in estrogen-dominant phases, leaving no doubt about the connection between H_2_S and estrogens (20, 105).

H_2_S-producing enzymes plays a vital role in intrauterine tissues by regulating Hcy levels and thus preventing pathological conditions. Uncontrolled Hcy levels can lead to hyperhomocysteinemia (73, 77) associated with various adverse outcomes in pregnancy, including impaired implantation (131), reduced litter size (124, 131), neural tube defects (132), miscarriages (5, 64), preeclampsia (5, 133, 134), hypertension (76, 134), and fetal growth restrictions (135). However, CSE is not a secondary enzyme in this matter. Is it also capable of generating H_2_S from Hcy and effectively regulating its levels (Figure 1) (74, 76, 77, 136, 137). Interestingly, *CBS-KO* in the uterus itself is not a direct cause of infertility in these individuals. Infertility in *CBS-KO* individuals occurs due to the resulting hyperhomocysteinemia or due to the action of another factor in the uterine environment of *CBS-KO* homozygotes. This indicates that the prominent role of H_2_S-producing enzymes during pregnancy is regulating Hcy levels around the growing fetus (64). It is worth noting that although *CBS-KO* may lead to reduced fertility or even infertility in female offspring, this is not the case for male offspring (64, 122, 131). It is possible that the effect is related to the pathways of female sex hormones, such as LH and E2, as described in previous sections, and may not necessarily affect male fertility. However, further research would be needed to confirm these assumptions.

Given that H_2_S-producing enzymes play a specific role during pregnancy, it can be assumed that the gas they produce also plays a role. In the context of previously mentioned pathogenic states, H_2_S likely inhibits the soluble fms-like tyrosine kinase-1 (sFlt1), a vascular endothelial growth factor (VEGF) antagonist associated with hypertension and preeclampsia (138). Because elevated Hcy levels are a risk factor for preeclampsia, H_2_S may also prevent the onset of preeclampsia through this pathway (18, 73, 133, 139).

#### 3.2.3 The role of H2S in placenta

Like the uterus, a reduced CBS expression towards the end of gestation has been described in placental and decidual tissues. H_2_S likely serves to maintain the integrity of the chorion/amnion before birth by slowing down the aging of the fetal membranes’ cells, so it is not surprising that its expression in these tissues decreases with the onset of labor (Figure 4) (18, 89). It is also interesting to note that both CBS and CSE expression in fetal membranes decreases much more during physiological labor than in infants delivered by cesarean section (17, 140). This shows that H_2_S is necessary for maintaining pregnancy, and a decrease in its expression appears to be one of the critical factors leading to the physiological onset of labor (16, 140). However, it should be noted that the role of H_2_S in the placenta may vary between species. For example, hypoxic conditions in the human placenta lead to increased H_2_S production, which is not observed in rat placenta (18). H_2_, S also contributes to proper placental development by promoting angiogenesis through placental growth factor (PIGF), VEGF, and signaling pathways PKB, nitric oxide synthases (NOS)/NO, and MAPK3/1 (141–143). VEGF is a key factor in regulating placental angiogenesis and this process is stimulated by activation of MAPK pathway in placental endothelial cells (144). However, in contrast to these positive effects of H_2_S, an association has been described between increased CBS expression in placentas and infants with Down syndrome, indicating that proper regulation of H_2_S expression in intrauterine tissues is crucial for physiologically ongoing gravidity (145–147).

The relationship between H_2_S and two other gasotransmitters in fetal membranes is intriguing. While the CO donor (hemin) in fetal membranes does not affect H_2_S production, the NO donor (sodium nitroprusside) leads to a significant increase in H_2_S production in this tissue (18). It is, therefore, possible that both H_2_S and NO synergistically contribute to maintaining the integrity of fetal membranes and pregnancy. This would imply that intrauterine tissues can be included among many other tissues where a mutual relationship between H_2_S and NO has been observed (112, 148–151).

## 4 The role of H_2_S during embryo development

For several years, it has been known that the human trophoblast produces H_2_S through the expression of CBS and CSE, with some studies indicating that CSE is the primary

H_2_S-producing enzyme in the first trimester of pregnancy (33, 143). Generally, supplementing the culture medium with H_2_S and NO donors supports embryo development *in vitro*. Once again, the synergy between these two gasotransmitters was described in this tissue, as H_2_S produced by the trophoblast, like VEGF, stimulates endothelial nitric oxide synthase (eNOS) activation (143). However, the precise role of these gasotransmitters in embryogenesis remains unclear. One of the main roles of H_2_S during embryo development is likely epigenetic regulation of embryogenesis, cell cycle, support of DNA formation, and proliferation (152, 153). Based on previous research confirming the regulation of specific promoters by H_2_S, for example, in vascular smooth muscle cells (154), it can be hypothesized that this regulation is also functional in mammalian embryo cells. This hypothesis is supported by research confirming that H_2_S modulates genes encoding proteins involved in early embryo epigenetic regulation (152). Even though the precise mechanism of embryonic epigenetic regulation by H_2_S is unknown, it can be assumed that H_2_S has a positive effect on early embryonic development, and it may even be essential for enhancing transcription and modification of specific embryonic genes related primarily to metabolism (33). Conversely, reduced expression of H_2_S-producing enzymes may lead, for example, to reduced PIGF production causing fetal growth restriction (FGR) or recurrent spontaneous miscarriages (124, 155).

Furthermore, H_2_S appears to be an important factor in transporting the morula from the oviduct to the uterus, as inhibition of CBS expression leads to embryo retention or prolongs its transport. H_2_S likely acts against contractile endothelins, facilitating oviduct peristalsis and, consequently, the transit of the embryo itself (83). H_2_S also promotes proliferation, migration, cytoskeleton remodeling, and invasion of trophoblast cells, where it activates various types of kinases (e.g., FAK, Src, ERK), Rho GTPases, and upregulates metalloproteinases 2 and 9 (89). On the other hand, excessive expression of CBS and CSE in the oviducts may be a sign of ectopic gravidity or embryonal carcinoma, so it cannot be conclusively stated that higher levels of CBS and CSE expression in this tissue indicate physiological embryo transport (83). Proper regulation of H_2_S expression is also essential in preventing the development of intrauterine growth restriction (IUGR) and preeclampsia (155). Additionally, H_2_S protects the heart of chicken embryos by regulating myocardial K_ATP_ channels (156).

H_2_S-producing enzymes have been identified even in zebrafish embryos, where there were described 2 *cbs* orthologs – *cbsa* and *cbsb* (157). *Cbsb* is crucial for ion homeostasis, while *cbsa* appears redundant (158, 159). These results indicate that H_2_S is essential in embryonic development across various taxa.

## 5 Conclusion

The production of H_2_S has been demonstrated in all female reproductive tissues, primarily through the enzymes CBS and CSE and, to a lesser extent, through 3-MST (Table 1). We can assume that H_2_S plays a crucial role in various physiological processes associated with female reproduction, given its ability to vasodilate uterine and umbilical vessels, as well as maintain pregnancy through both the tocolytic effects of H_2_S and its capability to preserve the integrity of fetal membranes. Additionally, H_2_S has anti-aging effects on mammalian oocytes, supporting their maturation and ovulation, aiding in the transport of early embryos into the uterus, and epigenetic regulation of their genes. Further on, an important characteristic of H_2_S-producing enzymes, CBS and CSE, is their ability to regulate homocysteine levels in the vicinity of cells through the production of H_2_S. This mechanism within the female reproductive tract serves to prevent pathological conditions such as hyperhomocysteinemia, which can lead to preeclampsia, miscarriages, congenital fetal abnormalities, and others.

Conversely, dysregulation of H_2_S signaling may be associated with various pathological conditions. It has been reported that aberrant H_2_S metabolism results in impaired oviductal transport of embryos and developmental delay of preimplantation embryos in mice (83). It has also been shown that dysregulated placental CBS/H_2_S signaling significantly contributes to increased embryonic resorption in mice (124). Notably, H_2_S production was found to be upregulated in the human oviduct in ectopic pregnancy, suggesting the involvement of dysregulation of H_2_S homeostasis (83). Dysregulation of H_2_S signaling has been also linked to the pathogenesis of preeclampsia (5, 140). Abnormal H_2_S signaling has recently been reported to be involved in diabetes-related uterine dysfunction as it was found that in non-obese diabetic mice, uterine H_2_S production is 2-fold higher than in the control group. This increase in H_2_S associated with 3-MST has been shown to cause a reduction in spontaneous endogenous uterine contractions (160). In addition, CBS has been proposed to promote ovarian cancer progression, tumor growth, and drug resistance (161), while CSE has been associated with breast cancer metastasis promotion (162).

The effects of H_2_S and subsequent signaling pathways in the aforementioned tissues are well-described. These effects are mediated by kinases (PKA, PKB, MAPKs), ion channels (T and L-type Ca^2+^, K_ATP_, BK_Ca_), transcription factors (NF-κB), and other cellular messengers (NO, E2, PIGF, cytokines). A particularly interesting function of H_2_S is its epigenetic effects, involving chromatin modification and activation of specific promoters, as well as its interaction with female sex hormones (LH, E2). Yet, these effects are not sufficiently elucidated, although clarifying their precise molecular aspects might result in the development of new methods and drugs, particularly in the field of women’s health and perinatal medicine.

In conclusion, a comprehensive understanding of H_2_S function could lead to its therapeutic use in disorders related to reproduction. For instance, its tocolytic and vasodilatory effects could be utilized to maintain pregnancy, support embryo implantation, and prevent miscarriages. The interaction of H_2_S with LH and E2 could also be used in the development of new drugs regulating the menstrual cycle or supporting superovulation in women undergoing oocyte aspiration prior to *in vitro* fertilization. H_2_S could also enhance the culture media of oocytes and embryos in IVF clinics, promoting their proper development and increasing the chances of successful pregnancy. Additionally, it may serve as an effective treatment for conditions like hyperhomocysteinemia, preeclampsia, or irregular estrus/menstrual cycles.

## Author contributions

AP: Conceptualization, Writing – original draft, Writing – review & editing. ZP: Conceptualization, Writing – original draft, Writing – review & editing. BK: Writing – review & editing. NZ: Writing – review & editing. EC: Writing – review & editing. PP: Supervision, Writing – review & editing. MS: Supervision, Writing – review & editing.

## Supplementary material

SUPPLEMENTARY TABLE 1 This file contains the original prompt in Czech and its translation into English. AI was used primarily as a guide for the translation, and our team edited and refined the resulting text.
