# Supplementary material for: Hydrogen sulfide and its role in female reproduction
Source: Front Vet Sci. 2024 Jun 12;11:1378435. doi: 10.3389/fvets.2024.1378435 (PMC11202402; doi:10.3389/fvets.2024.1378435)
Supplement: SUPPLEMENTARY TABLE 1 — This file contains the original prompt in Czech and its translation into English. AI was used primarily as a guide for the translation, and our team edited and refined the resulting text. [file Table_1.pdf]

PROMPT USED FOR TRANSLATION BY CHATGPT

Original prompt in Czech:

„Ahoj, můžeš mi přeložit akademický text do angličtiny?“

Translated prompt from Czech to English:

„Hi, can you translate an academic text into English for me?“
